# The Effect of Kinesiotape on Flexion-Extension of the Thoracolumbar Back in Horses at Trot

**DOI:** 10.3390/ani10020301

**Published:** 2020-02-13

**Authors:** Cajsa Ericson, Pernilla Stenfeldt, Aagje Hardeman, Inger Jacobson

**Affiliations:** 1 Animotion Rehab, Kalles ängsväg 1, 760 15 Gräddö, Sweden; cajsaericson@gmail.com; 2 Hästrehab i Ängelholm AB, Vanstadsv. 121, 262 91 Ängelholm, Sweden; pernilla.stenfeldt@telia.com; 3 Tierklinik Luesche GmbH, 49456 Luesche, Germany; aagje.hardeman@gmail.com; 4 Dep. of Equine Sciences, Faculty of Veterinary Medicine, Utrecht University, 3512 Utrecht, The Netherlands; 5 Dep. of Anatomy, Physiology and Biochemistry, Swedish University of Agricultural Sciences, 75007 Uppsala, Sweden; 6 Division of Health Science, Luleå University of Technology, 971 87 Luleå, Sweden; inger.jacobson@ltu.se

**Keywords:** horses, biomechanics, locomotion, activity, rehabilitation, physiotherapy, kinesiotape, kinesiology tape

## Abstract

**Simple Summary:**

Weak back muscles and back pain are commonly seen in horses today; these problems are often caused by poor training techniques and lead to other problems such as poor performance, lameness, and pain. Veterinary treatment and/or rehabilitation is a necessity. Kinesiotaping is a commonly used treatment method in equine physiotherapy sand veterinary rehabilitation. The method theoretically stimulates the sensory pathways from the taped region that in turn modulates the neuromuscular activity and locomotor function to improve locomotion and/or range of motion (ROM). The aim of this study was to determine if kinesiotape applied to the abdominal muscles, with the intention of activating them as back flexors, would affect the ROM in flexion-extension (sagittal plane) of the thoracolumbar back of the trotting horse. Eight horses, aged 5–15 years, were included and were trotted on a straight line, 2 × 30 m, with and without kinesiotape. The differences between the two conditions were measured by an optical motion capture using reflective markers placed along the thoracolumbar spine. No statistical significance was shown between the two groups of horses; although some horses showed individual changes indicating that kinesiotape may have affected those horses. The evidence base for rehabilitation and training methods is essential, and more research is needed to understand whether there is any potential benefit of the kinesiotape.

**Abstract:**

Kinesiotape theoretically stimulates mechanoreceptive and proprioceptive sensory pathways that in turn may modulate the neuromuscular activity and locomotor function, so alteration of activation, locomotion and/or range of motion (ROM) can be achieved. The aim of this study was to determine whether kinesiotape applied to the abdominal muscles would affect the ROM in flexion-extension (sagittal plane) in the thoracolumbar back of horses at trot. The study design was a paired experimental study, with convenient sample. Each horse was randomly placed in the control or the intervention group and then the order reversed. Eight horses trotted at their own preferred speed in hand on a straight line, 2 × 30 m. Optical motion capture was used to collect kinematic data. Paired *t*-tests, normality tests and 1-Sample Wilcoxon test were used to assess the effects of the kinesiotape. No statistical significance (*p* < 0.05) for changes in flexion-extension of the thoracolumbar back in trot was shown in this group of horses. Some changes were shown indicating individual movement strategies in response to stimuli from the kinesiotape. More research in this popular and clinically used method is needed to fully understand the reacting mechanisms in horses.

## 1. Introduction

Physiotherapy has become an important part of injury prevention and rehabilitation of neuromuscular, myofascial, and osseo-ligament dysfunction, pain and/or injuries including back and pelvic pain in the pleasure and the sport horse. Objective research to validate physiotherapeutic interventions and methods in the horse is a necessity for the field to advance.

During locomotion, the thoracolumbar spine/pelvic complex (which will be referred to as back from hereon) is very dynamic; hence there is a need for adequate intervertebral stability [1]. Both the epaxial (above the transverse processes) and hypaxial muscles (below the transverse processes) are involved in locomotion and stabilization of the spine and play an important role in the movement of the equine back [1,2].

Back problems are common clinical presentations of stiffness, gait abnormalities, temperamental changes, poor performance [3,4,5] and finding the right and differential diagnosis, treatment and rehabilitation of the equine back is challenging due to multifactorial causes [3,4,5]. During rehabilitation of back dysfunction, stability and movement control is dependent on appropriate contribution of the neuromuscular system [6]. The need for control and dynamic stabilization of the intervertebral segments of the back is essential during exercise and rehabilitation to recruit and strengthen hypaxial musculature [7].

Different methods and aids are used during training and rehabilitation of the back with the intention of strengthening and activating the muscles to make them work in a coordinated (and usually) sport specific manner. There is limited research on the effects of training aids on the back [6,8,9,10,11].

Kinesiotape is an adhesive tape made of cotton with an elasticity of 130%–140% of its neutral state. The tape is not restrictive like the traditional athletic or medical tapes and it provides support to muscles without limiting the ROM [12,13,14,15]. The use of kinesiotaping in human sports medicine has increased during the last decade. Its use is believed to improve a variety of neuromuscular disorders in humans; however research outcomes are not comparable due to different hypotheses, outcome measures and applications. [16,17]. Poor standardized methods when evaluating the effect makes a critical review difficult.

Kinesiotaping is also commonly used in veterinary physiotherapy during rehabilitation and injury prevention training. Theoretically the physiological effects may be transmittable to horses due to the similarities in neuromuscular and neuromotor control pathways [18,19,20,21,22,23]. The aim of using tape is to stimulate mechanoreceptive and proprioceptive activity in the skin, fascia, ligaments, and joints and thereby create a sensory afferent activity from the taped region. The equine skin is densely innervated with sensory nerves and receptors closely connected to the hair follicles [24] with a thinner epidermis compared to human skin [25]. The tape aims to affect the neuromotor control system and the coordinated relationship between neural and muscular activity thus achieving modulation or alteration of the activation, locomotion and/or ROM [2,7,12,26].

The authors are not aware of any published studies regarding the effect of the kinesiotape in horses. There is a need to increase the evidence base of the effect of commonly used rehabilitation- and training aids to be able to improve the quality of rehabilitation and training, to achieve the best possible results and thereby also increase equine welfare.

The aim of this study was to determine if kinesiotape attached to abdominal muscles would affect the dorsoventral (flexion-extension) ROM in the thoracolumbar back. We hypothesized that by applying kinesiotape to the abdominal muscles it is possible to stimulate them thus affecting the trunk system by either stabilizing and/or affecting the ROM in the thoracolumbar back.

## 2. Material and Methods

According to the ethical review committee of the German national regulations, this intervention was considered routine clinical practice and non-invasive, therefore no specific ethical approval was needed and informed consent was documented for all of the owners taking part in this study.

The data collection was conducted at Tierklinik Lüsche, Bakum, Germany. The horses were analyzed in an indoor arena with a soft surface mixture of sand and textile fibers (FairGround^®^), the arena was harrowed daily.

Eight horses of different genders, breeds (German warmblood, Friesian, and German riding pony), disciplines (jumping, dressage or both) and ages (5–15 years: mean value: 8.8 years) were used in the study. Inclusion criteria were horses assumed to be functionally sound by the owners and in regular work. The horses went through lameness evaluation by an experienced clinician before the tests started with presence of lameness graded 0–5/5 using the AAEP (American Association of Equine Practitioners) lameness scale [27] (Table 1). All horses underwent a clinical examination of the back by experienced animal physiotherapists where findings of higher muscular tone were graded 0–4 [28] (Table A1), and pain 0–3 [29] (Table A2).

Data was acquired via Qualisys Motion Capture Systems, with 20 high-speed infrared cameras, set to a sampling frequency of 100 Hz. Calibration was done daily before the start of the measurements, according to the manufacturer’s instructions. The average calibration residual was 3.2 mm.

Synchronized video recordings were obtained for each measurement (Sony HDR-CX330).

**Figure 1:**
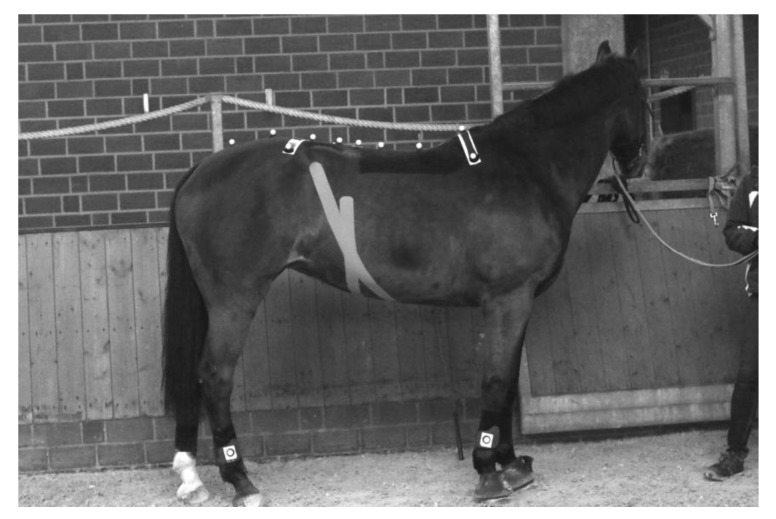
Position of the reflective markers on the horse at T6, T12, T15, T18, L3, L5, tuber sacrale, S3 and bilateral markers on T6 and tuber coxae. Please note that lateral markers of T12 and T15 are missing on the photo. Markers placed on the limbs were not used for analysis in this study.

Sixteen spherical and reflective skin surface markers, 25 mm in diameter, were placed with double-sided adhesive tape on the skin above the dorsal spinous processes of T6, T12, T15, T18, L3, L5, and S3, on the tuber sacrale and bilaterally on the tuber coxae (Figure 1). T6 had markers bilaterally at a fixed distance of 18 cm from the central T6-marker and segments T15 and T18 had two markers each bilaterally placed at the lateral rib angle, lateral to m. longissimus dorsi. Markers used in this study were T6, T12, T15, L5, L3 and the tuber sacrale.

The markers were placed after palpation of the anatomical landmarks by the same experienced animal physiotherapist in all horses (C.E). The positions of the markers were marked with a permanent marker pen so they could be placed in the same positions on subsequent days for the repeated measurements of untaped control.

Placement of the kinesiotape on the horse: across the abdomen at the level of the 16-18 ribs and across the aponeurosis of external oblique muscles from the xiphoid process of the sternum to the paralumbar fossa bilaterally.

VetkinTape*^®^,* a Dutch tape developed for animals, was used during the trials. The tape is 6 cm wide and was attached across the abdomen at the level of the 16–18 ribs and across the aponeurosis of external oblique muscles from the xiphoid process of the sternum to the paralumbar fossa bilaterally (Figure 1). The same animal physiotherapist attached the tape with no tension/stretch and rubbed the tape for heat activation of the adhesive. During all data acquisitions the tape was not observed to lose adherence or peel away from the horse’s skin.

Horses were presented with a normal stable halter except number 1, which was presented in a bridle with a snaffle bit. Prior to data collection, horses were walked for 5 min and trotted on the lunge for 5 min on each rein by the same person, then changed to a lead rope and markers and tape were applied. The same handler trotted up the horses in hand on a straight line and data was collected over a distance of 2 × 30 m without and with tape. For the trials the mean of measured strides per trial was 14.

Speed was calculated by smoothed differentiation of the horizontal coordinates (x, y) of the marker on the tuber sacrale.

**Figure A1:**
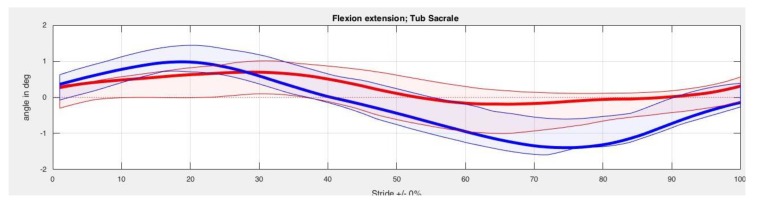

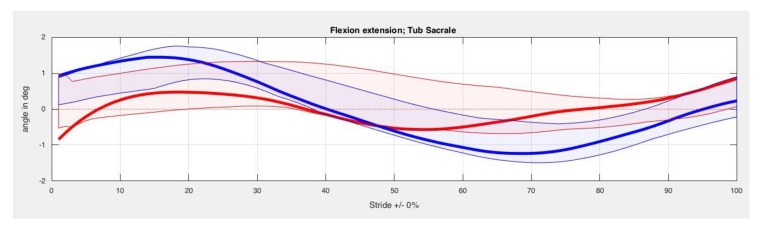
Flexion-extension angular patterns of the tuber sacrale of horse no. 4 (mean stride) at trot without kinesiotape (the upper graph) and with kinesiotape (the lower graph). The thickened line indicates the mean, shaded area the standard deviation. Left hindlimb red and right hindlimb blue.

**Figure A2:**
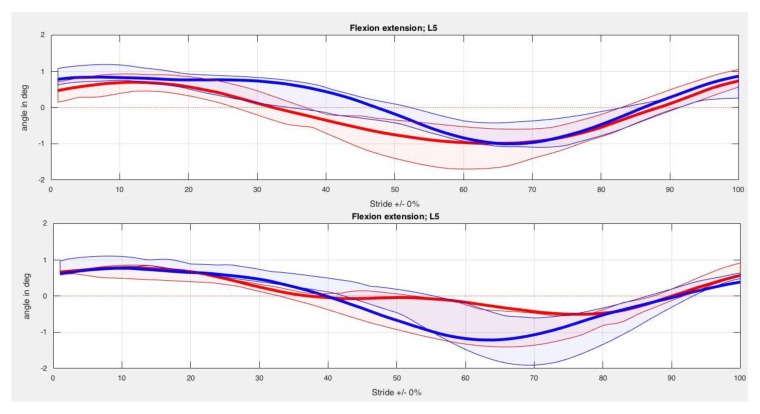
Flexion-extension angular patterns of L5 of horse no. 4 (mean stride) at trot without kinesiotape (the upper graph) and with kinesiotape (the lower graph). The thickened line indicates the mean, shaded area the standard deviation. Left hindlimb red and right hindlimb blue.

**Figure A3:**
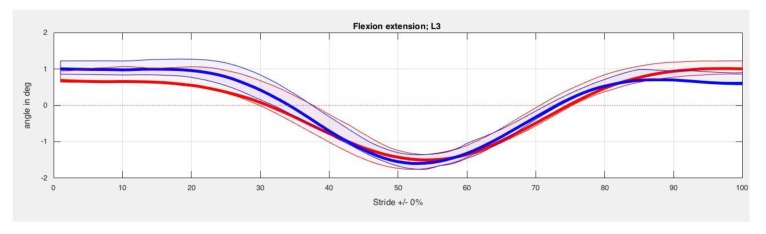

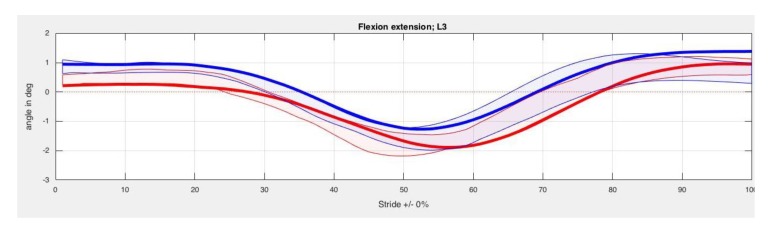
Flexion-extension angular patterns of L3 of horse no. 4 (mean stride) at trot without kinesiotape (the upper graph) and with kinesiotape (the lower graph). The thickened line indicates the mean, shaded area the standard deviation. Left hindlimb red and right hindlimb blue.

**Figure A4:**
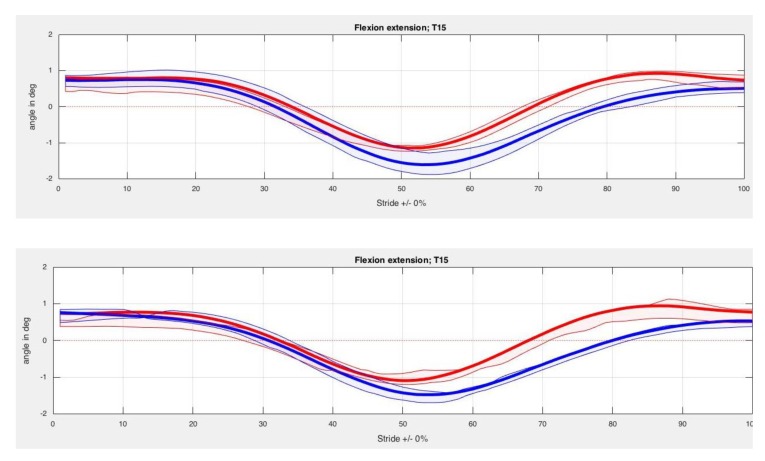
Flexion-extension angular patterns of T15 of horse no. 4 (mean stride) at trot without kinesiotape (the upper graph) and with kinesiotape (the lower graph). The thickened line indicates the mean, shaded area the standard deviation. Left hindlimb red and right hindlimb blue.

**Figure A5:**
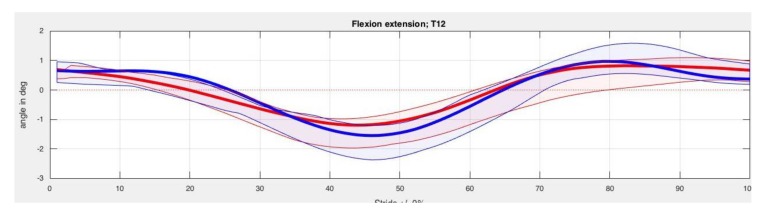

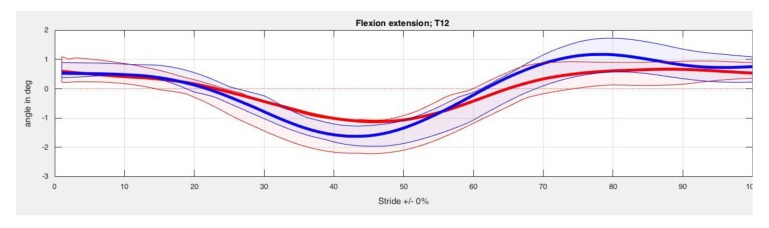
Flexion-extension angular patterns of T12 of horse no. 4 (mean stride) at trot without kinesiotape (the upper graph) and with kinesiotape (the lower graph). The thickened line indicates the mean, shaded area the standard deviation. Left hindlimb red and right hindlimb blue.

**Figure A6:**
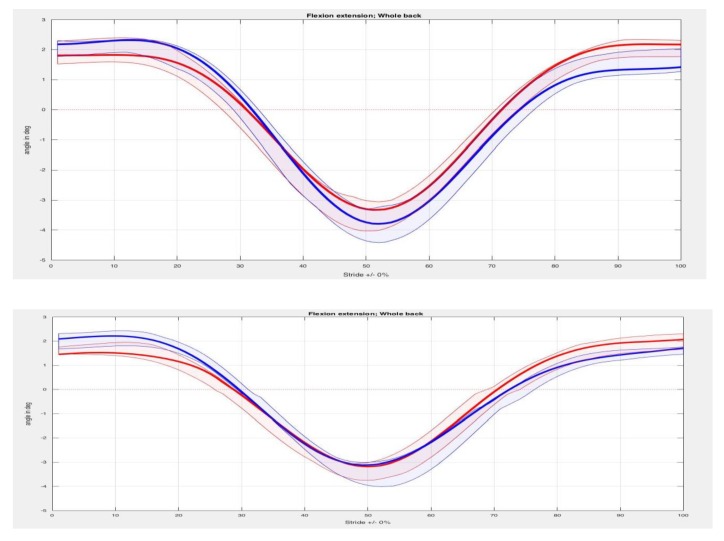
Flexion-extension angular patterns of the “whole back” of horse no. 4 (mean stride) at trot without kinesiotape (the upper graph) and with kinesiotape (the lower graph). The thickened line indicates the mean, shaded area the standard deviation. Left hindlimb red and right hindlimb blue.

The ROM in flexion-extension was calculated by proprietary software (Qhorse) between the actual segment and a straight line between the markers before and after trot up and the segments analyzed were T12, T15, L3 and L5. The “whole back” (WB) was calculated as the angle between the withers (T6 segment), the T15 segment and tuber sacrale (Figure A1, Figure A2, Figure A3, Figure A4, Figure A5 and Figure A6).

The statistical procedures were performed with IBM SPSS (Version 20.0, IBM SPSS Statistics for Windows) Armonk, NY: IBM Corporation.

Descriptive statistics were used to calculate means and standard deviations (SD). Due to the small sample size and non-normality in data distribution, Wilcoxon Signed Ranks Test was used to estimate differences between control and intervention group. Level of significance was set at *p* < 0.05.

## 3. Results

Mean speed was 3.2 m/s. Maximum lameness score was 1 out of 5 on the AAEP lameness scale [27] on all of the study days so no horses were excluded from the study. Muscular tone and pain reactions varied from 1–3 and 0–3 respectively [28,29] (Table 1).

When evaluating the effect of kinesiotape (across the abdomen) for all horses and segments on ROM of flexion-extension of the thoracolumbar back in trot, no statistically significant difference between the untaped and the taped horses was observed (Table 2). However some horses showed a tendency to increased flexion-extension. (Table 3). Horse number 6 showed a tendency to increased ROM in flexion-extension of the back in the L5 and L3 segments (total ROM left and right hindlimb). Horse number 5 showed slightly increased values in the L5 segment (both the maximum and the minimum ROM) and a tendency to raise the back (Table 3). The data were also analyzed for symmetry between left and right hindlimb during a full stride with no significant differences occurring (Table 3, Figure A1, Figure A2, Figure A3, Figure A4, Figure A5 and Figure A6).

## 4. Discussion

Using optical motion capture, this study aimed to establish the potential effect of kinesiotaping on spinal kinematics. No significant changes in flexion-extension of the back (neither segmentally nor in the whole back) across all horses and levels were seen. In two horses, a tendency to increased flexion-extension was observed during taping and this may indicate that the horse used individual strategies (the body’s response to a stimuli, conscious or unconscious that can change a movement pattern) when reacting to the stimuli of the kinesiotape. Neither of these two horses showed any lameness. One horse (number 6) showed values that differed from the others, when checking video recordings, all markers were in place throughout the tests and there were no signs of errors when troubleshooting the Qualisys system.

Optical motion capture is the current gold standard to measure 3D movement with a high degree of accuracy [30]. Skin-fixated markers are often used to collect kinematic data but skin displacement during kinematic analysis may have an effect when analyzing as the movement of the skin can cause errors in capturing data [31,32]. Comparing skin-fixated markers and bone-fixated markers placed at the segments of T6, T10, T13, T17, L1, L3, L5, and S3, data were satisfactorily determined in flexion-extension of thoracolumbar vertebrae in walk and trot [33]. Skin-fixated markers are non-invasive and provide the ability to perform analyses of locomotion without being restricted to a laboratory environment. The use of the 25 mm markers gives an increased precision and measuring errors are less than 3.5 mm (C. Roepstorff, Qualisys; personal communication). Furthermore, the marker positions were recorded with a permanent marker pen between the trials so the markers could be placed on the same positions the following day for the repeated measurements of untaped control.

There is less movement in flexion/extension in the thoracolumbar back on a straight line compared to lunging [34]. Also a greater flexion-extension and lumbosacral flexion is seen on the lunge when using other training aids [6]. This may be due to an increased activation of the trunk muscles and we may have achieved more differences in flexion-extension if we had chosen to lunge the horses instead of trotting them up on a straight line.

The activity of *m. rectus abdominis* is lower in walk than in trot [35] and horses increase the tension of their trunk muscles when increasing speed to stabilize the back and so reduce the range of flexion-extension [8,10,36,37]. When changing from walk to trot, the epaxial muscles are activated for sagittal plane stability and reciprocal activity of *m. rectus abdominis* is recruited [8]. Because of this antagonistic function between *m. longissimus dorsi* and *m. rectus abdominis* in trot [1,35,36,37,38] it may have been easier to detect differences in walk rather than in trot.

Muscle forces, transmitted by protraction and retraction of the limbs, will influence the pattern of motion of the back in axial rotation, lateral bending, and flexion-extension [33,39]. The head position will affect the back [40,41,42] and so will the gravitation pull on the abdominal contents [43,44]. As this system of myofascial and osseo-ligamentous structures is under continual tension, changes in one part of the system will affect the other in the movement of the back [39,45]. This study has not considered the influence of head and neck position; the horses in the study were presented on a halter and were free to choose their own head and neck position.

Kinesiotape is believed to affect the underlying muscles through the fascia that plays a role in the regulation of movement, transmitting tension and affecting body posture [46,47]. When comparing humans and horses, there are findings concluding almost similar functionally interconnecting myofascial kinetic lines in both species [47]. The application of the tape on the densely innervated equine skin may affect the different layers of soft tissue as they are all connected by fascia [48]. The *m. cutaneous trunci* attaches between the skin and fascia, covering the thoracic walls and unites with *mm. pectorals, latissimus dorsi* and *teres major* through fascial bands [49,50]. As *m. cutaneous trunci* responses to tactile stimulation of the skin [49] kinesiotape may be a method to consider when trying to activate the abdominal muscles. However, this study did not show any evidence of that a possible activation (from the kinesiotape) of the abdominal muscles affected the flexion-extension of the thoracolumbar back. When trying to address the abdominal muscles, a study was done with elastic resistance bands around the abdomen, the results showing an increased dynamic stability of the thoracolumbar back is not comparable with this study as they used elastic resistance band around the hind quarter in combination [11].

The sensory facilitation on the skin influences the neuromotor control: the coordinated relationship between neural and muscular activity [2]. The sensorimotor cortex is responsive to central and peripheral stimulation by mechanisms, important for motor learning and re-education in man [51] and thereby kinesiotape may induce changes in the cortex, but there is a need for further studies in kinesiotaping to be able to make this as a statement.

In this study, the tape was attached with no stretch or tension. Some practitioners attach the tape with up to 25%–50% stretch and some recommend no stretch. There are several schools in the technique of kinesiotaping; stretching the elastic tape from distal to proximal or vice versa over a muscle will, according to some practitioners, affect a muscle in an activating or relaxing way. To standardize the use of the tape, we decided to attach the tape without stretch. Stretching the tape may have given an increased sensory input due to the increased tension of the tape.

The horses in this study were considered functionally sound by their owners and were in regular work. In the muscular examination, different levels of pain reactions and high muscular tone in thoracolumbar muscles were present in most of the horses. Equine back dysfunction may cause muscle weakness and/or spasm and a general restriction of ROM that may alter the posture, flexibility, stability, and proprioception in a negative way. The subsequent alteration or suboptimal biomechanics or joint dysfunction affects the normal neuromuscular function and can create changes in segmental vertebral ROM [3,4,8,52,53,54,55]. Muscle score assignment in clinical examination and back kinematics are related and scoring the muscles may be of benefit when identifying horses with poor stability in the back [56]. The relationship between increased muscle tone and pain may have affected the ROM in the horses in our study and interestingly, the horses with reaction on palpation or who had high muscular tone were the same horses that showed minor asymmetries in the subjective evaluation. Studies have shown a relationship between back pain and lameness supporting a high prevalence (74%) of lameness in horses with back pain [57].

The abdominal muscles, except for supporting the abdominal viscera, mainly perform the active flexion of the thoracolumbar spine and limit thoracolumbar extension during the stance phase through its simultaneous activity with *m. longissimus dorsi* [36,37,45]. The movement of the back is, however, reduced because of the antagonistic function between *mm. longissimus dorsi* and *rectus abdominis* in trot [1,35,36,37,38]. Together with the epaxial stabilizers, the deep abdominal muscles provide a proactive stability and control of the back [8]. Kinesiotape has been suggested to potentially be an effective method when re-educating and activating an appropriate neuromotor control and function [48], but in this study we could not see any significant changes of flexion-extension, nor signs of stabilization of the thoracolumbar back.

The intersegmental motion of the back is relatively small; the ROM in flexion-extension (T6–S3) varies between 2.8–4.9 degrees in trot [33]. Haussler et al. [58] described the ROM in flexion-extension in more detail: T14–16: 0.7–1.1, L1–L3: 0.2–0.4 and L6–S3: 1.5–3.3 degrees. This study showed only small individual changes in flexion-extension between the untaped and taped condition, but the values were too small to show statistical significance. The horses in this study were not homogenous in age, breed, disciplines, or levels of competition. Horses 5, 6, and 8 showed slightly more changes in flexion-extension in the lumbar area than the other horses and when looking at these three horses, they were younger than the others. One thought is that young horses, with less trunk stability and strength, may be more affected in flexion-extension ROM with the kinesiotape, they may also have been more sensitive to the stimulation of the taped area of the skin.

Muscle activity adjacent to the individual vertebrae was not assessed in this study. Electromyographic analysis of the abdominal and deep epaxial muscles during activity would provide a more complete understanding of the eventual effect of kinesiotape on muscular activity and it may describe the effect that veterinarians and physiotherapists believe they are visualising; a clear increased contraction of the abdominal muscles when taped, and what the rider is feeling; “he is rounding up”, “he is lifting his back”, “he feels more stable and straight” and “he is working more from behind”.

It may also be of interest to evaluate the potential effect of the kinesiotape after a period of training with the kinesiotape, as similar muscle activation exercises (dynamic mobilization) and mobilization techniques have shown to increase the cross-sectional area of the m. multifidi [1,38,59,60].

In this study, the number of horses used was low restricting any further conclusions from the results. Power is increased by a paired design but would have required 12 in each group to detect a 20% difference in angles (assuming standard deviation of 25%) power 80% with 95% confidence.

## 5. Conclusions

This study did not show any significant effect of kinesiotape in ROM in extension-flexion or stabilization of the back of the trotting horse. Some small changes were shown indicating individual movement strategies in response to stimuli from the kinesiotape. There is a need for more research in this clinically and commonly used method to fully understand the mechanisms behind it in horses, and there is a need for further studies of rehabilitation- and training aids to achieve evidence-based methods to rehabilitate or train the horses in the best possible way and to improve horse welfare.

## Figures and Tables

**Table 1:** Horses in the study presented in age, breed, gender (Wbl = Warmblood), discipline (J = Jumping, D = Dressage), AAEP lameness scale (0 = lameness not perceptible/5= minimal weight bearing in motion and/or at rest) [27], findings in muscle tone (0 = Hypotonicity/4 = Severe increased muscle tone) [28] and pain reactions (0 = Pain free/3 = Severe pain response) [29] at palpation of the back.

Horse	Age	Breed	Gender	Discipline	Lameness Scale 0–5	Muscle Tone 0–4	Pain Score 0–3
1	9	Wbl	Mare	J	RF 1	2	2
2	12	Wbl	Mare	J	RF 1	1	2
3	9	Wbl	Mare	J	0	2	2
4	9	Wbl	Gelding	D	0	1	1
5	6	Pony	Mare	J/D	0	1	1
6	5	Wbl	Gelding	J/D	0	2	2
7	15	Wbl	Gelding	D	RF 1	3	3
8	5	Friesian	Gelding	D	0	1	0

**Table 2:** Mean, SD and *p*-values for all horses and back segments. Tuber Sacrale (TS), 5^th^ Lumbar segment (L5), 3rd Lumbar segment (L3), 15th Thoracic segment (T15), 12th Thoracic segment (T12) and “The whole back” (WB) in degrees in ROM (Range of motion); Kinesiotape and Control group.

Back Segment	Kinesiotape Mean (SD) in Degrees	Control Mean (SD) in Degrees	*p*-Value
TS Max left	20.20 (3.91)	20.34 (3.74)	0.33
TS Min left	17.78 (3.38)	18.05 (3.43)	0.40
TS ROM left	2.42 (1.07)	2.29 (1.17)	0.40
TS Max right	20.50 (3.30)	20.50 (3.54)	0.89
TS Min right	18.29 (3.13)	18.47 (3.49)	0.78
TS ROM right	2.21 (0.71)	2.03 (0.85)	0.89
L5 Max left	0.18 (6.31)	1.52 (2.74)	0.67
L5 Min left	−1.99 (7.48)	−0.35 (2.90)	0.33
L5 ROM left	2.16 (1.31)	1.86 (0.66)	0.67
L5 Max right	0.20 (5.84)	1.45 (2.58)	0.89
L5 Min right	−2.35 (7.39)	−0.36 (2.71)	0.78
L5 ROM right	2.56 (1.72)	1.80 (0.40)	0.16
L3 Max left	1.23 (5.74)	−0.75 (2.47)	0.48
L3 Min left	−1.35 (5.44)	−2.92 (2.38)	0.58
L3 ROM left	2.57 (1.01)	2.17 (1.16)	0.58
L3 Max right	1.42 (6.01)	−0.78 (2.54)	0.58
L3 Min right	−1.49 (4.84)	−3.15 (2.49)	0.89
L3 ROM right	2.91 (1.47)	2.37 (1.18)	0.29
T15 Max left	−5.65 (2.19)	−5.81 (2.07)	0.33
T15 Min left	−8.06 (2.50)	−7.91 (2.23)	0.89
T15 ROM left	2.41 (0.70)	2.11 (0.90)	0.26
T15 Max right	−5.60 (2.15)	−5.71 (2.18)	0.89
T15 Min right	−7.96 (2.21)	−7.65 (1.97)	0.29
T15 ROM right	2.37 (0.69)	1.94 (0.85)	0.40
T12 Max left	−13.84 (3.25)	−13.73 (2.48)	0.78
T12 Min left	−16.21 (3.55)	−15.91 (2.78)	0.78
T12 ROM left	2.37 (0.72)	2.17 (0.92)	0.61
T12 Max right	−13.37 (3.26)	−13.55 (2.59)	1.00
T12 Min right	−16.04 (3.60)	−15.76 (2.81)	0.58
T12 ROM right	2.66 (0.57)	2.21 (0.89)	0.26
WB Max left	0.37 (0.33)	0.31 (0.34)	0.18
WB Min left	−0.05 (0.13)	0.02 (0.35)	0.74
WB ROM left	0.43 (0.27)	0.29 (0.26)	0.21
WB Max right	0.35 (0.31)	0.32 (0.30)	0.55
WB Min right	−0.07 (0.14)	0.00 (0.37)	0.89
WB ROM right	0.42 (0.26)	0.32 (0.23)	0.67

**Table 3:** Differences in degrees in ROM of flexion-extension (flex-ext) between taped and non-taped runs in eight horses at five segments (Tuber Sacrale (TS), 5th Lumbar segment (L5), 3rd lumbar segment (L3), 15th Thoracic segment (T15), 12th Thoracic segment (T12) and “The whole back” (WB), no statistical significance was shown across all horses. Left/Right Max/Min are the symmetry parameters for the vertical displacement that show the differences between the two extremes of the movement, each step results in one upward and one downward movement between the right and left halves of a stride. Values in bold shows the tendencies of increased values.

Horse and Segment	Flex-Ext, ROM, Left Max in Degrees	Flex-Ext, ROM, Left Min in Degrees	Flex-Ext, Left ROM in Degrees	Flex-Ext, ROM, Right Max in Degrees	Flex-Ext, ROM, Right Min in Degrees	Flex-Ext, Right ROM in Degrees
1. TS	−0.21	0.32	−0.53	0.19	1.00	−0.81
1. L5	0.42	0.54	−0.13	−0.09	0.34	−0.43
1. L3	−0.09	0.06	−0.16	0.03	0.07	−0.04
1. T15	0.11	0.03	0.08	−0.31	−0.32	0.01
1. T12	−1.41	−2.03	0.62	−1.45	−0.95	−0.50
1. WB	0.05	0.01	0.04	−0.02	0.00	−0.01
2. TS	0.38	−0.06	0.44	−0.32	−0.04	−0.29
2. L5	−0.66	−0.49	−0.17	−0.37	−0.76	0.39
2. L3	0.27	0.15	0.12	0.17	−0.10	0.27
2. T15	0.45	0.32	0.13	0.27	0.32	−0.05
2. T12	0.91	2.07	−1.16	0.73	0.98	−0.25
2. WB	0.00	0.05	−0.05	−0.03	0.08	−0.11
3. TS	−0.23	0.13	−0.36	0.31	0.46	−0.16
3. L5	−0.05	−0.18	0.13	−0.22	−0.04	−0.18
3. L3	0.72	−0.19	0.91	0.31	0.06	0.25
3. T15	−0.10	−0.46	0.36	−0.31	−0.71	0.40
3. T12	−1.34	−1.54	0.21	−1.23	−2.00	0.76
3. WB	0.10	0.00	0.10	−0.04	−0.02	−0.02
4. TS	−0.73	−0.79	0.06	0.21	0.51	−0.30
4. L5	0.13	0.56	−0.43	−0.02	−0.14	0.11
4. L3	0.01	0.09	−0.07	0.49	0.74	−0.25
4. T15	−0.05	0.20	−0.24	0.03	0.31	−0.29
4. T12	0.02	−1.36	1.38	0.66	−0.27	0.94
4. WB	0.03	0.02	0.01	0.00	0.03	−0.02
5. TS	−0.63	−1.58	0.95	−1.24	−2.24	1.00
5. L5	1.41	1.30	0.11	1.76	1.39	0.37
5. L3	− 2.90	−2.20	−0.70	−3.06	−2.96	−0.10
5. T15	0.89	1.36	−0.47	0.76	1.20	−0.44
5. T12	0.66	−1.48	−0.22	−1.41	−1.08	−0.33
5. WB	−0.16	0.11	0.21	0.22	0.14	0.08
6. TS	0.11	−0.33	0.22	−0.62	−0.74	0.21
6. L5	−13.09	−16.37	3.28	−12.50	−17.37	4.88
6. L3	17.26	16.73	0.52	18.88	16.32	2.56
6. T15	−0.89	−1.81	0.92	−0.29	−1.87	1.58
6. T12	0.66	1.90	−1.25	0.84	1.34	−0.50
6. WB	−0.16	−0.10	−0.06	0.05	−0.10	0.15
7. TS	0.57	0.55	0.02	0.69	0.48	0.21
7. L5	0.63	0.55	0.08	1.16	0.68	0.49
7. L3	−0.37	−0.21	−0.16	−0.32	−0.24	−0.08
7. T15	0.21	0.11	0.10	−0.16	−0.34	0.18
7. T12	−0.47	−0.48	0.01	0.24	−0.87	1.11
7. WB	0.05	0.04	0.02	0.04	0.04	0.01
8. TS	−0.13	−0.33	0.20	0.83	−0.81	1.64
8. L5	0.48	0.98	−0.50	0.32	−0.07	0.39
8. L3	0.92	−1.85	2.77	1.07	−0.62	1.69
8. T15	0.65	−0.92	1.58	0.92	−1.07	1.99
8. T12	2.52	0.53	1.99	3.02	0.63	2.39
8. WB	0.12	−0.73	0.85	−0.01	−0.77	0.76

## Appendix A

**Table A1:** Muscle tone scoring system.

Muscle Tone Score	Classification	Description
0	Hypotonicity	Loss of muscle tone
1	Normal	Normal, expected muscle tone for a horse standing square
2	Mild	Mildly to moderately increased muscle tone
3	Moderate	Moderately to severely increased muscle tone
4	Severe	Severely increased muscle tone

**Table A2:** Pain scoring system.

Pain Score	Classification	Description
0	Pain free	No reaction
1	Mild	Nose wrinkling, ear flattening, slight spasm on palpation without associated movements
2	Moderate	Head jerk, teeth bearing, tail lashing, stamping foreleg, aggressive tail flattening, rising hind leg, spasm on palpation with associated local movement (i.e., Pelvic tilt)
3	Severe	Kicking, biting, rearing, sour attitude, restless, sinking away from the hand
